# Knowledge, attitudes and beliefs toward polypharmacy among older people attending Family Medicine Clinic, Nairobi, Kenya

**DOI:** 10.1186/s12877-024-04697-9

**Published:** 2024-02-05

**Authors:** Maureen Kamau, Njeri Nyanja, Adelaide M. Lusambili, Jacob Shabani, Gulnaz Mohamoud

**Affiliations:** 1 Department of Family Medicine, Aga Khan University Hospital, Nairobi, Kenya; 2 Institute for Human Development, Aga Khan University, Nairobi, Kenya

**Keywords:** Older people, Sub-saharan Africa, Polypharmacy, Knowledge, Attitudes, Beliefs

## Abstract

**Background:**

Life expectancy has increased over the last century among older people, particularly those aged over 60 years. Aging is associated with increased disability, multiple chronic conditions, and increased use of health services managed with polypharmacy. There are few studies on polypharmacy and aging in sub-Saharan Africa, and it is unclear what older people know and their attitudes toward polypharmacy. This paper presents findings from a study that aimed to understand older people’s knowledge, attitudes and beliefs about polypharmacy.

**Methods:**

A qualitative study using in-depth interviews of 15 patients aged 60 years and older who were taking more than five medications per day. The study was conducted at the Family Medicine Clinic (FMC), Aga Khan University Hospital, Nairobi. Data were analyzed using NVivo 12 software.

**Result:**

Majority of participants had a good understanding of their underlying health conditions, but they did not know the specific names of the medications they were taking. Participants had diverse attitudes toward polypharmacy, with both positive and negative perceptions. Although adverse side effects were reported, participants remained positive because they believed these medicines were beneficial. Religion, faith and living healthy lifestyles were perceived to contribute to their positive attitude toward polypharmacy. Stigma and the cost of medication were reported as barriers.

**Conclusion:**

This study provides valuable insights into the complexities of polypharmacy in older people. It highlights the importance of patient education, fostering strong patient-provider relationships, de-stigmatization, and improving medication affordability and accessibility. Further research could explore the polypharmacy of older people attending public institutions in rural Kenya.

**Supplementary Information:**

The online version contains supplementary material available at 10.1186/s12877-024-04697-9.

## Introduction

The World Health Organization (WHO) estimates that the world’s population of those over 60 years will rise to 22% by 2050 with 80% of this population coming from low and middle-income countries [1]. In 2019, approximately 2 million of the Kenyan population was aged 60 years and above [2]. This is projected to double by 2050 [3]. Health related expenditure is set to escalate due to increase in life expectancy and the population of older peoplei [4]. Older people have an increased prevalence of multimorbidity [5]. The WHO has acknowledged that polypharmacy prevalence is set to escalate with an increase in chronic disease [6]. Advanced chrpnic conditions such as chronic kidney disease is associated with polypharmacy [7]. In the US, the older the person the worse the degree of polypharmacy [8]. In South Africa, increased pill burden defined as more than 10 pills per day,is responsible for the multiple adverse effects experienced among older patients [9]. An Ethiopian study showed that polypharmacy led to poor quality of life [10]. Additionally, older people form a vulnerable sector of our society so it is imperative to have health related care guidelines to address this unmet need [11, 12]. It is imperative that a concerted and well coordinated global effort is required to offer efficient health care systems to abate polypharmacy among older patient with multiple chronic conditions [13].

Quantitative studies have been conducted in Africa on the effects of polypharmacy [10, 14]. The assessment of older patients knowledge, attitude and belief on polyphamcy using qualitatitative methods has mostly been done outside Africa [15, 16]. A South African a qualitatitative study [9], explored the knowledge, attitudes and beliefs of older persons about polypharmacy. African studies on polypharmacy have elucidated on the effects of polypharmacy from the prescribers rather than the patients perspective. In Nigeria, the importance of health care worker intervention in addressing factors responsible for polypharmacy was emphasized [14]. A South African study found that the knowledge of older people about their conditions and medications was enhanced by a few health workers who educated them [9].

Since patient centred perspective from Kenyan cultural context on polypharmacy is scarce, we posit our study to cover this gap.

## Methodology

### Study design

This was a qualitative study using in-depth interviews.

### Study setting

The study was conducted at the Aga Khan University Hospital - Nairobi (AKUH-N). The hospital is a private teaching and referral hospital in East Africa that mainly caters to upper -and upper-middle-class patients in Kenya. This study was conducted at the Family Medicine Clinic (FMC) at AKUH-N, a well-established hospital clinic with approximately 375 patients in attendance every week. Approximately 20% of patients attending the FMC are older than 60 years.

### Study population

#### Inclusion criteria

Participants included patients older than 60 years, who were taking more than five medications per day, who were capable of communicating in English or Kiswahili, and who had been attending the FMC.

#### Exclusion criteria

Patients with cognitive impairment, such as dementia and psychosis, and those who were identified as too ill, that is, Glasgow Coma Scale of less than 15, patients who were hemodynamically unstable as depicted by their vital signs and who fell within Canadian Emergency and acuity Scale (CETAS) of one, two or three, were excluded from the study.

### Sampling process, sample size and consent

Participants were recruited using purposive sampling. A total of 15 participants were recruited for the study.

The participants were approached during regular clinic hours at the FMC between 8 am and 4 pm on Mondays and Fridays. During the triage process, the principal investigator, with the help of the triage nurses at the FMC, confirmed the age of the patients and enquired about the number of medications taken daily. Those who took more than five medications and fulfilled the inclusion criteria were verbally invited to participate in the study. A study brief was provided verbally during the invitation process and explained in simple English or Kiswahili (Language was as per the patient’s preference). If the patient agreed to participate, they were invited to provide written consent. A mutually convenient time was then decided upon for the interview, either at the same sitting or later at the FMC.

During the interview, the researcher explained the study information focusing on the purpose, benefits, possible risks and confidentiality clause. Participants read the consent forms, and those who agreed to participate in the study provided written informed consent.

The participants were inviited to ask any questions that may have arisen.

### Data collection

MK (the lead researcher) conducted interviews after undergoing training and familiarizing herself with the interview guide. Interviews were conducted at the FMC at a time convenient and acceptable to the participants in one of the offices. Informed consent procedures were conducted out at the start of each interview, and participants were given ample opportunity to make inquiries before the interview. Participants were also reminded of their willful withdrawal.

An open-ended and flexible topic guide encouraged free-flowing dialog with participants. The interviews were conducted in English or Kiswahili (language choice was based on participants’ preference) and audio-recorded with participants’ verbal and written consent. Two interviews were conducted in Kiswahili, and the rest were conducted in English; transcription of the two Kiswahili interviews was first performed and the transcripts were later translated into English. Field notes were also taken during the interview process.

The study tool was piloted by MK on three participants, and transcribed and reviewed by AL, GM and NN (all of whom were co-investigators). Challenges such as older people’s understanding of various terminologies were discussed. The team agreed that during the interview, several prompts and synonyms would give the participants more time to delve into their feelings. The lead researcher and co-investigators carefully carefully crafted the interview guides to include a broad range of sub- topics such as perceptions and influence of polypharmacy, knowledge and sources of information, attitudes, beliefs, barriers and coping strategies. Participants were interviewed once and only after signing written informed consent. At the end of each interview, a debriefing statement was read to all participants. The study was conducted in the context of COVID-19. All infectious disease precautions, were strictly followed.

Participants were asked questions about their knowledge and understanding of polypharmacy, beliefs, storage and attitudes toward the medication. Interviews were audio recorded, carefully labeled and transferred to a secured laptop at the FMC. Of the 15 interviews, two were conducted in Kiswahili and translated from Kiswahili to English. All interviews were transcribed by Sharon Ochieng, a research assistant. GM and NN validated transcribed data by listening to the audio and translating the narratives. A unique participant study identification (PTID) was allocated to the participants to identify their study records.

### Data analysis

**Fig. 1:**
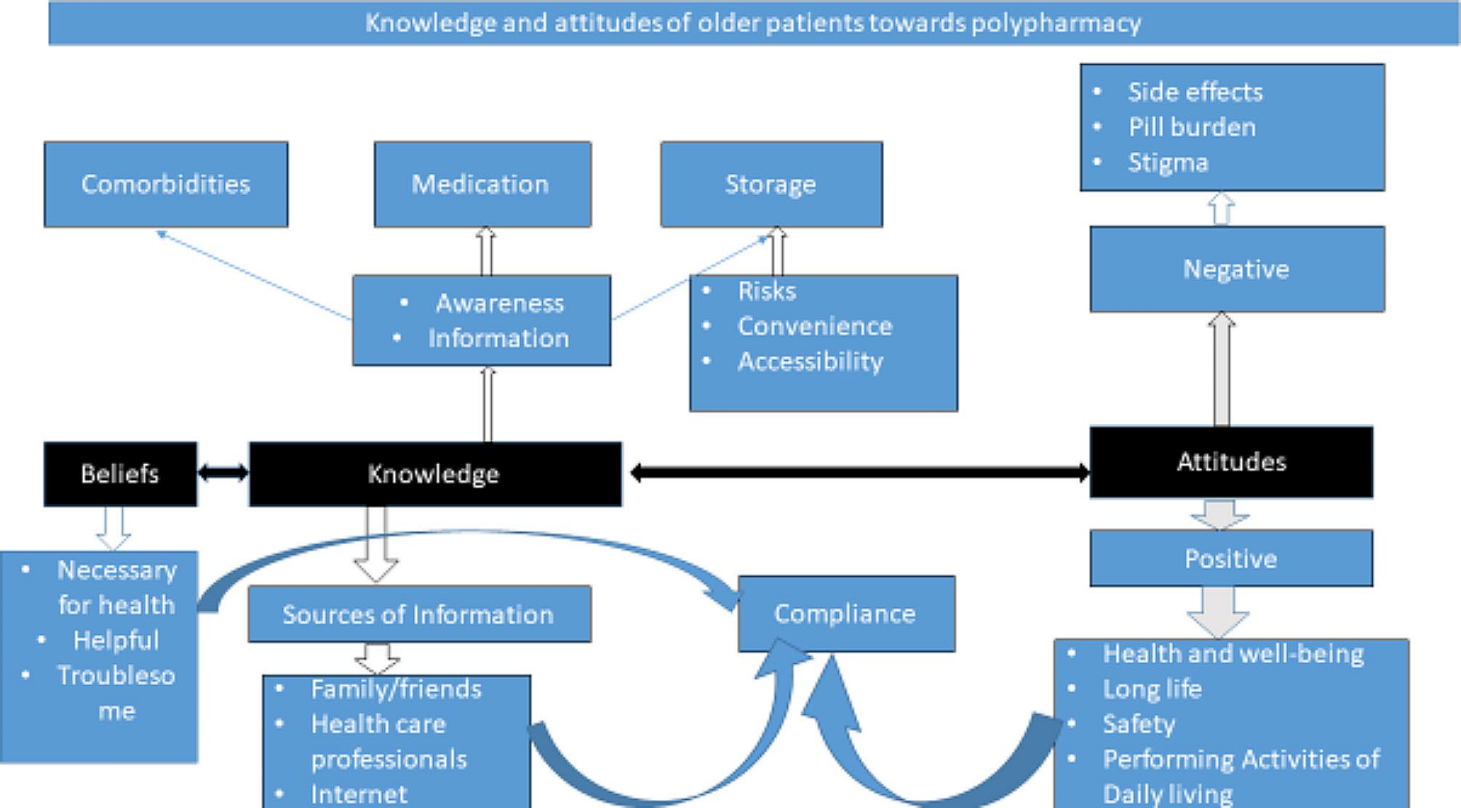
Thematic summary

A thematic analysis process was conducted using the six steps approach developed by Braun and Clarke (2006) [17, 18]. The researchers familiarized themselves with the data and generated the initial codes by dividing the data into small meaningful chunks. All authors reviewed the codes, and developed a codebook. Researchers frequently met to discuss the data by creating a matrix that mapped all the themes as summarized below, in Fig. 1.

Member checking was performed after data analysis, where research findings were summarized, and transcripts were shared with the participants to verify the data.

In this study saturation was characterized by comprehensiveness and depth of the data as well as the replicability of the data when no new information was forthcoming [19]. This was achieved after interviewing 12 patients.

## Results

We interviewed 15 patients. Data from the first three patients were used for pilot interviews. The findings presented here are from 12 patients, four males and eight females, aged between 60 and 74 years. All the participants had more than one comorbidity and took more than five different types of medications. The patient demographics are summarized in Table 1.

**Table 1:** Patients demographics

PATIENT	GENDER	AGE	OCCUPATION	RELIGION	RACE	MARITAL STATUS	NO.OF MEDICATIONS	LIST OF COMORBIDITIES
1	Female	74	Businesswoman	Christian	African	Married	5	1,5,6
2	Male	65	Civil servant	Christian	African	Married	5	1,2
3	Female	67	Businesswoman	Christian	African	Married	5	1
4	Female	64	Judge	Christian	African	Single	6	1,2
5	Male	69	Mechanic	Muslim	Indian	Divorced	5	1,2,9
6	Male	61	Civil engineer	Christian	African	Married	7	1,2,3
7	Male	73	Businessman	Christian	African	Married	5	1,2,3
8	Female	65	Nurse	Christian	African	Separated	5	1,2
9	Female	74	Farmer	Christian	African	Married	5	1,2,3
10	Female	67	Housewife	Muslim	African	Married	5	1,2
11	Female	67	Administrator	Christian	African	Single	5	1,8
12	Female	62	Lawyer	Christian	African	Widow	5	1,2,3,4,7

Key:

1- Hypertension

2- Diabetes

3- Dyslipidaemia

4- Osteoarthritis

5- Asthma

6- Low back pain

7- Gastritis

8- Epilepsy

9- Coronary artery disease

The results are presented by the themes generated in this study.

### Knowledge and information

Participants demonstrated an understanding of the medicines they were taking for their comorbidities but had limited knowledge about the medication names.

*“In the morning, I take two medications, one for sugar and one for pressure. Then, in the evening, I will take three medications. There are two for cholesterol and, uh, the other for blood* [pressure] *and the other for sugar. So in a day, I take [seven] 7 tablets.” (61-year-old male).*

Many participants emphasized the importance of safe medication storage, especially when living with family members. They took measures to ensure that their medications were securely stored.

*“I have created space in my wardrobe where I put them* [medicine] *so when I get the medicine…because of convenience, I will take them early in the morning or the evening. Second, they are my medicines, so in the sitting room, they* [may get] *into the wrong hands because I do not live alone. I have a family, so somebody can misuse them* [the medication].” (65-year-old male).

Sources of knowledge and information on their health condition, medications they took, and general management included health professionals, family and friends and the internet.

*…when I was first diagnosed, the doctor and the healthcare providers explained it to me. “This is a life-long condition…” they explained. When it came to diabetes which was two years ago, and it was discovered here again… the healthcare providers took me through. I was shown the diet, the exercise, the medicine and how often, how regularly I should take them.”* (65-year-old male).

Furthermore, oneparticipant reported using the internet to research their conditions and medications,.

*“Mmmh. I am always very keen. Whatever I am given, I Google… the side effects and all that…I normally Google I want to know more about* [the medications.” (74-year-old female).

### Experience and attitudes toward polypharmacy

Participants in the study had both positive and negative perceptions of polypharmacy. Their attitudes were influenced by their relationships with healthcare providers, family members, and broader social support networks. Many participants believed that the multiple medications they took daily were essential for their well-being and had a positive attitude toward their conditions and prescribed medications.

*“They* [medications] *are of benefit to me, but they are many*.” (65-year-old male).

*“… I made up my mind to be positive a long time ago. In addition, uh, I said the condition has come. There is nothing I can do about it. I can only cooperate with the doctors. I do not believe in witchcraft. I rely on the doctors whom God has given me. In addition, so I have lived my life”* (62-year-old female).

Most of the older participants interviewed reported that the medications they were prescribed enabled them to effectively perform activities of daily living (ADL), which showed that these medications managed their various comorbidities. One

*“This is why I can walk. I can do my own thing. This is why you see me at 74. “…They are just okay because if I do not take them, I would feel… I rarely fail to take. Because I was told by Doctor xxx that this is your food, keep it there when you are taking your food. I consider it my food. Therefore, I’m glad to take them.”* (74-year-old female).

Negative attitudes toward polypharmacy were often related to pill burden and concerns about long-term side effects.

`*"…what they do now to my body because of the, you know, cocktails. That is my concern because if my liver is not used to such kind of strong* [medications] *what… then there must be a problem there that comes with that. Yeah. “*… *“My body, my internal system. Might be interfered by that*.” (61-year-old male).

Despite these concerns, the participants understood the importance of medication adherence for their overall well-being and continued life.

*“And why am I disciplined? It is because it is my life. Yes. In fact, what I do is…in fact it is a bit awkward. If I do not take them religiously as I do or as per the doctor’s instruction, I see a stroke and a coffin. That is what I have in my mind. Yes, and because I am active and have a lot of projects for my family, I would want them completed. I know, yes, it is a burden. I’m told that medicines have side effects, but I am saying that those side effects can occur in the future when I am still healthy. Yes.*… *However, I know if I do not do this, something worse can happen. Therefore, which is better? Let me take my medicine religiously.”* (65-year-old male).

### Barriers and coping mechanisms

Some participants reported experiencing stigma related to their chronic illnesses, which was worsenedd by the need for polypharmacy. They often chose not to disclose their health conditions to close family members and friends, in line with cultural preferences.

*“I’m not taking more than what I have just told you, not even outside. Uh, it can help you lead a normal life because like my relatives do not know I’m even sick, that I have a condition…Nobody. In addition, even my children did not know until the other day, I do not talk about my sickness with them because I’m okay…So it helps.” (6*7-year-old female).

*“…but children, we do not keep telling them…As Africans, we do not want to keep talking about it”.* (65-year-old male)

The high cost of medications was another significant barrier, with some participants finding the expenses unaffordable.

*“When I retire, I have to admit to meet the costs* [of the medication] *myself, the drugs are quite expensive.”* (67-year-old female).

Among the coping mechanisms reported by the patients were praying and practicing religion, herbal therapies, and adopting a healthy lifestyle. These strategies served as emotional and physical support for managing comorbidities effectively.

*“The Bible and* [I] *hope that one day there would be no one sickness….having the hope that they will go away keeps you going.”* (65-year-old female).

Diet and exercise are common coping mechanisms used to manage conditions such as diabetes and high cholesterol, contributing to improved well-being and quality of life.

*“… I noticed that on those days when I go for exercises I am active, and I go for exercises, I breathe better… I feel lighter and stronger. Uh, other than when I get home and everything’s right there. Um, I feel more tired all the time. Yeah.”* (62-year-old female).

## Discussion

This study explored the knowledge, attitudes, and beliefs about polypharmacy among older people.

### Knowledge and information

The participants in this study had a good understanding of their underlying health conditions, but there was a gap in their knowledge of the specific names of the medications they were taking. Despite this gap, it is crucial to note that their lack of knowledge about medication names did not deter them from adhering to their prescribed regimens. This may indicate a level of trust in the healthcare system and emphasize the importance of patient education. The role of healthcare workers in educating older people has been reported in the literature [7, 9]. As the population of older people continues to increase in LMICs, there is a need to increase and train community pharmacists to reach out and educate older people on the usefulness and side effects of polypharmacy [20]. In addition, polypharmacy increases drug to drug interactions, hence the importance ofcoordination of care that can be provided by specialists such as family physicians [21].

Sources of knowledge, information and support included health professionals, friends, family, and colleagues. Our findings show that this is some of the challenges associated with polypharmacy. A relationship exists between individuals’ literacy levels and their adherence to medications [22]. Although most of our patients were literate, older people in low- and middle-income settings who cannot read and write may be worse off and even at a greater risk of nonadherence particularly if they are not aware of the medications they are consuming and of the potential side-effects. Therefore, healthcare professionals should prioritize patient education and ensure that patients not only understand their health conditions but also the names, dosages, and purposes of their medications. Providing clear, accessible information to patients may empower them to take a more active role in their treatment [23].

### Experiences and attitudes toward polypharmacy

Participants in this study had diverse attitudes toward polypharmacy, with both positive and negative perceptions. The relationships they had with healthcare providers, family members, and social support networks significantly influenced their attitudes. Many participants believed that the multiple medications they were prescribed were essential for their well-being and had a positive attitude toward their conditions and medications. The side effects from medication created a negative attitude toward polypharmacy, resulting in medication discontinuation in some patients in the present study, which was not reported to their physicians. This is similar to a South African study in which older people expressed concerns about moremedications that were responsible for the multiple adverse effects they experienced [9]. The study findings hence call for prescription review by healthcare workers at every clinical encounter.

Some patients had positive attitudes toward the medications, believing tthat were necessary for their well-being and survival. Our findings were similar to those in a Swedish study that found that patients were more compliant with medication that provided symptomatic relief [24].

Therefore, healthcare professionals should recognize the importance of patient- provider relationships in shaping patients’ attitudes toward polypharmacy. A positive and collaborative patient- provider relationship can lead to better treatment adherence and improved health outcomes.

### Barriers and coping mechanisms

Stigma, the cost of medications, and geographic challenges were identified as significant barriers to the effective management of polypharmacy. To mitigate this type of stigma, healthcare professionals should create supportive, non-judgmental environments that encourage open communication about health conditions.

Some participants chose not to disclose their health conditions because of stigma, which highlights the need for destigmatizing chronic illnesses. A Danish systematic review paper examined older people’s experiences with polypharmacy and recognized self-stigma [25]. In this study, for Muslims and Christians alike turning to religion through prayer was a coping mechanism. Studies that exploring the coping strategies that older people use to for deal with polypharmacy are limited. A Ghanaian study confirmed that religion and prayer are integral to ensuring continued medication use and follow up [22]. Our study was conducted during the COVID-19 pandemic, and most older people could not continue with their regular care at the FMC. A number of them opted for prescription refills rather than attending the FMC to receive their care. Some older people also use supplements to prevent COVID-19 infections, increasing the pill burden [26].

### Cost

Participants in our study found high cost of medication to be an important concern. Similarly, a study from Ethiopia found that 67% of older people felt that they had invested heavily in medications [10]. With rising living costs, many pensioners plunge into financial hardships and are unlikely to afford health care costs alongside other demanding needs [27].

Policymakers and healthcare providers should also work to reduce the financial burden of medications, especially for older adults.

### Study strengths andlimitations

This study used a qualitative approach to explore the perceptions of patients in a private healthcare facility in Kenya, Participants were selected using purposive sampling. The study’s strength is that the findings revealed the potential to improve healthcare among older people by highlighting the plight of polypharmacy among older people in Kenya. The insights gaiined from this study has important clinical implications for healthcare professionals and policymakers.

The study was performed in a private, high-end institution among affluent patients and focused on patients from a private rather than public institution,who were more likely to afford medical care and treatment. Interpretation of such findings in populations from less affluent settings or those seen at a public healthcare facility may differ This study is additionally limited by recall bias becuase we relied on participants’ recollections of their experiences.Kenya is a country with 45 different tribes and even more dialects. This study excluded patients who did not speak English or Kiswahili and may not be representative of the non-English and non-Swahili speaking Kenyan population. Nonetheless, our results represent a section of the population and may be helpful in influencing policies on geriatric care in Kenya and beyond, even though they cannot be generalised.

## Conclusion

In conclusion, this study provides valuable insights into the complexities of polypharmacy in older people. This study highlights the importance of patient education, strong patient- provider relationships, de-stigmatization, and improving medication affordability and accessibility. Addressing these factors can contribute to an effective and improved quality of life for older people managing polypharmacy.

## Recommendations

There is a need for ensuring optimum older patient education and awareness during the patient’s clinical encounter consultation to establish an understanding of their condition and the expected medication side effects. Fostering a better relationship among older people, their healthcare providers, family and friends may play an integral role in their adoption of attitudes and beliefs that enhance their overall health and well being thereby reinforcing medication adherence. Additional research that explores the knowledge, attitudes and beliefs of the geriatric population attending public institutions within the country may better enhance our understanding of the perceptions of polypharmacy among older people in Kenya. There is also a need for such studies in the resource limited and rural populations.

### Electronic supplementary material

Below is the link to the electronic supplementary material.

Supplementary Material 1

## Data Availability

The datasets generated or analyzed during the current study are not publicly available because of the confidential nature of the data as we interviewed 15 patients and the population of older people attending FMC is not large. Thus, someone familiar to the clinic including healthcare workers could easily identify their names if the transcripts are made public. The datasets are available from the corresponding author upon reasonable request.

## Declarations

### Disclaimer

There are no disclaimers.

## References

[1] World Health Organization. Ageing and health [Internet]. 2017 [cited 2023 Dec 31]. Available from: https://www.who.int/news-room/fact-sheets/detail/ageing-and-health.

[2] United Nations Population Fund. The State of Kenya Population [Internet]. 2020 p. 52. Available from: https://kenya.unfpa.org/sites/default/files/pub-pdf/state_of_kenya_population_report_2020.pdf.

[3] Margaret M, Mwaila, Mona T, Yousif (2022). Kenya’s ageing population: current and future prospects. World J Adv Res Rev.

[4] United Nations. Ageing Related Policies and Priorities in the Implementation of the 2030 Agenda for Sustainable Development. [Internet]. 2018. Available from: https://www.un.org/Development/Desa/Ageing/Wp-Content/Uploads/Sites/24/2019/07/Analysis-Ageing_vnrs_final28122018.pdf.

[5] He Z, Bian J, Carretta HJ, Lee J, Hogan WR, Shenkman E (2018). Prevalence of multiple chronic conditions among older adults in Florida and the United States: comparative analysis of the OneFlorida Data Trust and National Inpatient Sample. J Med Internet Res.

[6] World Health Organization. Medication safety in polypharmacy: technical report [Internet]. 2019 [cited 2023 Dec 31]. Available from: https://www.who.int/publications-detail-redirect/WHO-UHC-SDS-2019.11.

[7] Al-Mansouri A, Hamad AI, Al-Ali FS, Ibrahim MIM, Kheir N, Al-Ziftawi NH (2023). Pill-burden and its association with treatment burden among patients with advanced stages of chronic kidney disease. Saudi Pharm J SPJ off Publ Saudi Pharm Soc.

[8] Young EH, Pan S, Yap AG, Reveles KR, Bhakta K (2021). Polypharmacy prevalence in older adults seen in United States physician offices from 2009 to 2016. PLoS ONE.

[9] Naidoo K, van Wyk J (2019). What the elderly experience and expect from primary care services in KwaZulu-Natal, South Africa. Afr J Prim Health Care Fam Med.

[10] Tegegn HG, Erku DA, Sebsibe G, Gizaw B, Seifu D, Tigabe M (2019). Medication-related quality of life among Ethiopian elderly patients with polypharmacy: a cross-sectional study in an Ethiopia university hospital. PLoS ONE.

[11] Langmann E (2023). Vulnerability, ageism, and health: is it helpful to label older adults as a vulnerable group in health care?. Med Health Care Philos.

[12] United Nations. World Population Ageing [Internet]. 2017 p. 46. Available from: [https://www.un.org/en/development/desa/population/publications/pdf/ageing/WPA2017_Highlights.pdf](https://www.un.org/en/development/desa/population/publications/pdf/ageing/WPA2017_Highlights.pdf).

[13] Mangin D, Bahat G, Golomb BA, Mallery LH, Moorhouse P, Onder G (2018). International Group for Reducing Inappropriate Medication Use & Polypharmacy (IGRIMUP): position Statement and 10 recommendations for action. Drugs Aging.

[14] Akande-Sholabi W, Adebusoye L, Olowookere O. Polypharmacy and Factors Associated With Their Prevalence Among Older Patients Attending a Geriatric Centre in South-West Nigeria. West Afr J Pharm [Internet]. 2018 [cited 2023 Dec 31];29(1). Available from: https://papers.ssrn.com/sol3/papers.cfm?abstract_id=3508232.

[15] Schöpf AC, von Hirschhausen M, Farin E, Maun A (2018). Elderly patients’ and GPs’ perspectives of patient-GP communication concerning polypharmacy: a qualitative interview study. Prim Health Care Res Dev.

[16] Stefanacci RG, Khan T (2017). Can Managed Care Manage Polypharmacy?. Clin Geriatr Med.

[17] Braun V, Clarke V (2006). Using thematic analysis in psychology. Qual Res Psychol.

[18] Guest G, Bunce A, Johnson L (2006). How many interviews are enough? An experiment with data saturation and variability. Field Methods.

[19] Morse JM. Qual Health Res. 2015;25(5):587–8. Data were saturated. 10.1177/1049732315576699 25829508

[20] Beuscart JB, Petit S, Gautier S, Wierre P, Balcaen T, Lefebvre JM (2019). Polypharmacy in older patients: identifying the need for support by a community pharmacist. BMC Geriatr.

[21] Berkelmans PG, Berendsen AJ, Verhaak PF, van der Meer K (2010). Characteristics of general practice care: what do senior citizens value? A qualitative study. BMC Geriatr.

[22] Atinga RA, Yarney L, Gavu NM (2018). Factors influencing long-term medication non-adherence among diabetes and hypertensive patients in Ghana: a qualitative investigation. PLoS ONE.

[23] Turner JP, Currie J, Trimble J, Tannenbaum C (2018). Strategies to promote public engagement around deprescribing. Ther Adv Drug Saf.

[24] Moen J, Antonov K, Larsson CA, Lindblad U, Nilsson JLG, Råstam L (2009). Factors Associated with multiple medication use in different age groups. Ann Pharmacother.

[25] Eriksen CU, Kyriakidis S, Christensen LD, Jacobsen R, Laursen J, Christensen MB (2020). Medication-related experiences of patients with polypharmacy: a systematic review of qualitative studies. BMJ Open.

[26] Nwanaji-Enwerem JC, Boyer EW, Olufadeji A, Polypharmacy, Exposure (2021). Aging populations, and COVID-19: considerations for Healthcare Providers and Public Health Practitioners in Africa. Int J Environ Res Public Health.

[27] Santibáñez-Beltrán S, Villarreal-Ríos E, Galicia-Rodríguez L, Martínez-González L, Vargas-Daza E, Ramos-López E (2013). Economic cost of polypharmacy in the elderly in primary health care. Rev Médica Inst Mex Seguro Soc.

