# Supplementary material for: Knowledge, attitudes and beliefs toward polypharmacy among older people attending Family Medicine Clinic, Nairobi, Kenya
Source: BMC Geriatr. 2024 Feb 5;24:132. doi: 10.1186/s12877-024-04697-9 (PMC10845745; doi:10.1186/s12877-024-04697-9)
Supplement: Supplementary file 1 — Supplementary Material 1 [file 12877_2024_4697_MOESM1_ESM.docx]

## Appendix 1: Participant information About the Study

Study Title:

What is the knowledge, attitude and beliefs of towards polypharmacy amongst older people attending Family Medicine Centre (FMC) at the Aga Khan University Hospital Nairobi, Kenya (AKUHN)?

Principal Investigator:

Dr Maureen Kamau.

Supervisors:

Dr Gulnaz Mohamoud

Dr Adelaide Lusambili

Dr Njeri Nyanja.

Introduction

My name is Dr Maureen Kamau; a Masters's student in the field of Family Medicine at Aga Khan University Hospital, Nairobi.

I am currently carrying out research looking at the knowledge, attitudes and beliefs of older people towards polypharmacy at the Family Medicine Centre, Aga Khan University Hospital Nairobi, Kenya.

I am interested in the knowledge, attitudes and beliefs of older people towards polypharmacy.

Purpose of this study

This study aims to provide insight into the knowledge, attitudes and beliefs of geriatric patients towards polypharmacy.

Benefits of the study

The study will help establish health care practitioners understand the burden of polypharmacy on the older population. The outcome of this study will be important in providing this information to other health care workers at the Aga Khan University Hospital and in other institutions in Kenya. This will lead to an overall improvement of care for older people.

Risks/side effects

This study will involve older people who attend the Family Medicine Centre, Aga Khan University Hospital Nairobi.

There are no direct risks or side effects in partaking in this study.

Voluntary participation

Participation in this study is voluntary meaning you can choose to participate or not, and you are free to withdraw your participation in this study at any time.

Confidentiality

The information obtained from this research will be confidential and only accessible to the PI. Your information will be coded using non-identifiable numbers that is it cannot be traceable back to the patient.

Conduct of Study

Once consented, the in-depth interviews will be conducted at a suitable time and location for the participant.

**Appendix 2: Interview Guide**

IDENTIFICATION NUMBER:

___

AGE in years:

___

SEX:

Male ___ Female

RESIDENCE:

___

ETHNICITY:

___

OCCUPATION:

EDUCATION LEVEL:

No formal Education __Primary Education __Secondary Education __ Tertiary Education

MARITAL STATUS:

Single ____Married ____Widowed ____Separated ____ Divorced

RELIGION:

Christian ___ Hindu ____Muslim ___Other (Specify) _____

NUMBER OF MEDICATIONS:

____

NUMBER AND LIST OF CO-MORBIDITIES:

(list)

KEY QUESTIONS PROBES

What is the current knowledge about polypharmacy?

How many medications are you taking?

• What medications do you take?

• What medical conditions do you take the medication for?

• How do you store your medications?

Probe- Do you have a pillbox to store pills?]

• Do you use other medication alongside your prescribed meds? If so, which ones? And why

[Probes- Do you use herbal medications, supplements?]

• How many times in a day do you take each of the medications you have mentioned? Are they all taken at the same time?

[Probes- Who supervises the taking of drugs?

• How do you know when to take each of the medications?]

• Who has prescribed the medication you are taking?

[probes- how many doctors do you visit? Where do you collect the medication from?]

• How often does your doctor review your condition or medication with you? How do you know when you are due for review? Does your doctor tell you?

• Do you have any other information about the drugs you are taking? And where did you get the information from?

[Physician? Pharmacy? Family? Friend?]

What is your attitude about polypharmacy?

• Could you tell me your thoughts/feelings when you are taking these medications?

[Probes –how do you feel when you are in the process of taking the medicines

-Do you feel safe or like they may harm you?

-Do you have any concerns about the medications?

-Do you think all the medications are necessary for your health?

• What are your views about the medication you are taking?

[ probes - Do you think they are of benefit- if so, why?

If not- explain]

• What are some of the challenges you face in taking these medications?

• How do you cope with taking so many drugs?

What is your belief about polypharmacy?

• From the information, you shared about your views on the use of multiple medications (polypharmacy) overall what do you think about these multiple medications?

Probes are they helpful? are they troublesome? Are they an inconvenience?

How does it affect your daily activity?

• What prompts/ drives you to seek alternative/ additional medication?

• Is such a drive founded on the belief that the current medication is not working or that additional medication will make the treatment more effective?

We have come to the end of our interview. Do you have any other thoughts or ideas you wanted to share during this interview that you haven’t had the chance to?

Thank the participant for their time and for sharing this information with you.

**Appendix 3: Consent Form**

TITLE:

Knowledge, attitude and beliefs towards polypharmacy amongst older people, attending Family Medicine Clinic (FMC), at the Aga Khan University Hospital, Nairobi, Kenya (AKUHN).

Dear Sir/Madam,

Written Consent Statement

I have read the preceding information, or it has been read to me. I have had the opportunity to ask questions about it and they have been answered to my satisfaction. I consent to participate.

Name-

Contact-

• I agree to participate (please tick) • I do not agree to participate (please tick)

• I agree for a recoding to be used during _________________’s participation (please tick)

• I do not agree to _____________________________’s participation (please tick)

• I agree that the study material can be used for publication_______________(please tick)

Respondent’s Signature: ___________________________________________________

Date: __________________________________________________________________

For any enquiries, kindly contact the investigator on xxx.
